# Hysteretic Behavior of Proprotein Convertase 1/3 (PC1/3)

**DOI:** 10.1371/journal.pone.0024545

**Published:** 2011-09-15

**Authors:** Marcelo Y. Icimoto, Nilana M. Barros, Juliana C. Ferreira, Marcelo F. Marcondes, Douglas Andrade, Mauricio F. Machado, Maria A. Juliano, Wagner A. Júdice, Luiz Juliano, Vitor Oliveira

**Affiliations:** 1 Departamento de Biofísica, Universidade Federal de São Paulo, São Paulo, Brazil; 2 Ciências Exatas e da Terra, Universidade Federal de São Paulo, São Paulo, Brazil; 3 Centro Interdisciplinar de Investigação Bioquímica, Universidade de Mogi das Cruzes, São Paulo, Brazil; University of South Florida College of Medicine, United States of America

## Abstract

The proprotein convertases (PCs) are calcium-dependent proteases responsible for processing precursor proteins into their active forms in eukariotes. The PC1/3 is a pivotal enzyme of this family that participates in the proteolytic maturation of prohormones and neuropeptides inside the regulated secretory pathway. In this paper we demonstrate that mouse proprotein convertase 1/3 (mPC1/3) has a lag phase of activation by substrates that can be interpreted as a hysteretic behavior of the enzyme for their hydrolysis. This is an unprecedented observation in peptidases, but is frequent in regulatory enzymes with physiological relevance. The lag phase of mPC1/3 is dependent on substrate, calcium concentration and pH. This hysteretic behavior may have implications in the physiological processes in which PC1/3 participates and could be considered an additional control step in the peptide hormone maturation processes as for instance in the transformation of proinsulin to insulin.

## Introduction

Mammalian subtilases of yeast-kexin type are called proprotein convertases (PCs) or subtilisin-like proprotein convertases (SPCs) [1]. PCs are calcium-dependent proteases responsible for the processing/maturation of precursors proenzymes, neural and hormonal peptides, serum proteins, growth factors and surface glycoproteins of pathogenic bacteria and viruses [2], [3]. This family of peptidases is composed by PC1/3, PC2, furin, PC4, PACE4, PC5/6, PC7/8, SKI-1 and NARC-I [4]. Furin, PACE4, PC5-B, and PC7 are preferentially expressed in the constitutive secretory pathway, whereas PC1/3, PC2, and PC5-A are active in the secretory granules of the regulated secretory pathway of endocrine and neuroendocrine tissues [5].

All PCs have in tandem disposition the following structures: N-terminal signal peptide followed by profragment region, conserved subtilisin-like catalytic domain, conserved P-domain and divergent C-terminal tail [4]. PC1/3 initiates the formation of the active forms of neuropeptides by cleaving the precursor proteins after pairs of basic residues [6], the same general and conserved motif recognized by the majority of PCs [2]. PC1/3 is synthesized as a 753-amino acid zymogen [7], [8] and undergoes autocatalytic intramolecular processing of its N-terminal profragment in the ER [9], [10]. The generated 87-kDa protein is targeted to the regulated secretory pathway where it is further shortened by removal of 135 amino acids of its C-terminal tail, resulting in the 66-kDa form [8]. This C-terminal cleavage occurs at the dibasic Arg-Arg^617–618^ site, possibly by an autocatalytic event and this tail has been proposed to play a role in sorting of PC1/3 to the regulated secretory pathway [7]. The reported pH assays showed that both 87 and 66-kDa forms have higher activity in acidic environment [11] and along the secretory pathway PC1/3 finds an environment that pH decreases from 6.7 to 5.5 [12]. It also known that in the secretory granules calcium concentration can raise up to milimolar concentrations [13], [14]; and the peptidase activities of 87 and 66-kDa forms of PC1/3 are increased in calcium ion concentrations range 1 to 20 mM [11]. PC1/3 was reported to present complex enzymatic kinetics for the hydrolysis of substrates [15], and a lag phase (pre-steady-state) in the initial 8 to 10 minutes followed by a linear phase with a constant velocity of hydrolysis (steady-state) was reported [11].

**Figure 1:**
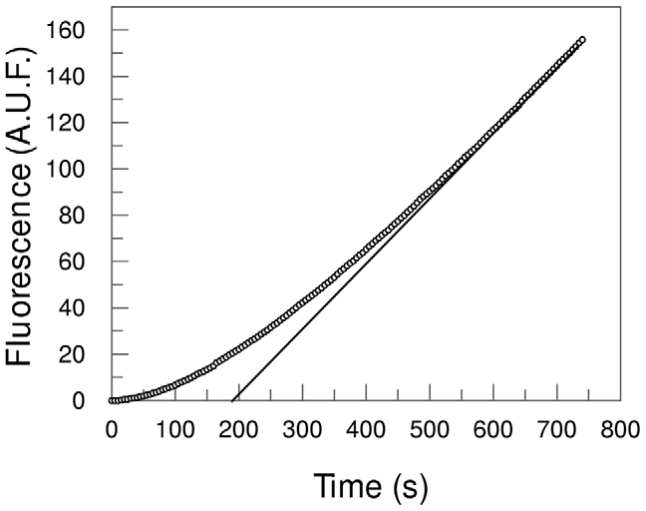
Time course of pERTKR-MCA (pE = pyrogluthamyl and MCA = 7-amino-4-methyl coumarin) hydrolysis by 66-kDa mPC1/3. The MCA release increases with time and the exponential transient phase (*lag* phase) precedes the linear steady-state phase. Hydrolysis conditions: mPC1/3 = 20 nM, Buffer = 20 mM BisTris, 20 mM CaCl_2_, pH 6.0 at 37°C. A.U.F. = Arbitrary Units of Fluorescence.

The aim of the present paper was to explore the unusual lag phase observed in the time course of activity of mPC1/3 as showed in Figure 1 for hydrolysis of the commercially available fluorogenic substrate pERTKR-AMC. In addition to this peptide we also examined the hydrolysis of fluorescence resonance energy transfer (FRET) peptides based on the PC1/3 natural consensus cleavage sites (indicated by the arrows): Abz-YTPKSRR↓EVED-Q-EDDnp, (proinsulin: NP_032412), Abz-SPREGKR↓SYSM-Q-EDDnp (POMC: EDL01384) and Abz-SKRSRR↓SVSV-Q-EDDnp (Japanese encephalitis virus polyprotein: AAT00231) [Abz, o*rtho*-aminobenzoic acid; Q-EDDnp, glutamyl-*N*-(2,4-dinitrophenyl) ethylenediamine]. The last FRET substrate was selected from a series of sixty substrates previously designed for furin studies [16]. The influence of calcium ion concentration and the pH on mPC1/3 activity lag phase were also examined. Finally, we verify if the mPC1/3 possesses the same uncomom kinetic behaviour with the larger substrate human salivary histatin-3, a histidine-rich peptide with 32 amino that was reported to be a substrate for PC1/3 [17]. Histatin 3 was synthesized, and the exclusive cleavage at the carboxyl side of R^25^ (DSHAKRHHGYKRKFHEKHHSHRGYR^25^↓SNYLYDN) by mPC1/3 was analyzed.

## Materials and Methods

### Enzyme preparation

**Figure 2:**
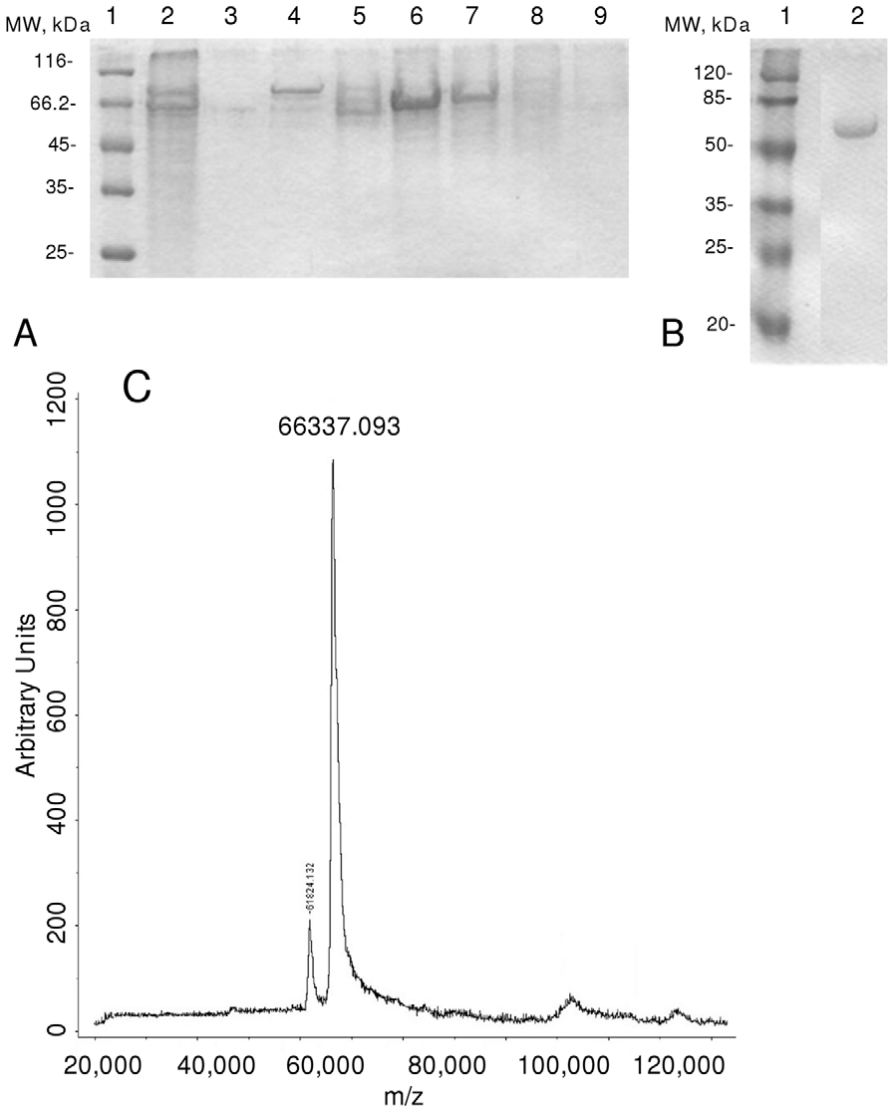
SDS-Page 12% of recombinant 66-kDa mPC1/3 purification. Panel A - Fractions resulting from Mono Q chromatography; gradient from buffer A (20 mM Bis-Tris, 2 mM CaCl2, 0.02% NaN3, 0.4 mM octylglucoside, pH 7.0) to 0–100% of buffer B (buffer A plus 1 M sodium acetate). Column 1: molecular weight marker; column 2–9: fractions from 0–100% of buffer. Pure 66-kDa enzyme was obtained in column 6 (10% of buffer B). Panel B - SDS-Page of pure 66-kDa best visualized (Column 2); Column 1: molecular weight marker. Panel C - MALDI-TOF analysis of purified mPC1/3.

Mouse PC1 was produced by overexpression in CHO cells using the dihydrofolate reductase-coupled amplification method. The expression and purification procedure were earlier reported [18], but several modifications were made. We used motionless T-75 bottles (Corning Life Sciences, Lowell, MA, USA) instead of roller bottles in order to reduce cell lysis during expression and thus the contaminants. Each expression gives a different proportion of 87, 74 and 66-kDa mPC1/3 forms, and many expressions were conducted until the pool obtained had the form of 66-kDa as the major component of the medium, according to SDS-PAGE analysis. The pooled conditioned medium was filtered through a 0.25 µm filter (Millex), diluted 1∶3 in buffer A (20 mM Bis-Tris, 2 mM CaCl_2_, 0.02% NaN_3_, and 0.4 mM octylglucoside, pH 7.0) and then subjected to Mono Q anion-exchange column chromatography (GE-Life Sciences, Piscataway, NJ, USA). Elution was accomplished with a gradient of buffer A to buffer B (buffer A plus 1 M sodium acetate). Figure 2-A shows the sodium dodecyl sulfate polyacrylamide gel electrophoresis (SDS-PAGE) 12% stained with silver of the pool formed by each peak of absortivity at 280 nm obtained from the Mono Q anion-exchange chromatography. The pool number 6 contained the homogeneous mPC1/3 as showed in the SDS-PAGE in Figure 2-B. The matrix assisted laser desorption ionization/time of flight (Maldi-TOF) spectrum in Microflex-LT (Bruker-Daltonics, Billerica, MA, USA) was obtained from this fraction (Figure 2-C). Protein batches eluted with a homogeneity >95% were stored at −80°C in 20% glycerol and used in all subsequent analyses. Protein concentrations were determined by the Bradford dye-binding assay using bovine serum albumin as standard [19].

### Peptides

Highly sensitive FRET peptides were synthesized by solid-phase procedures, as described elsewhere [20], [21]. All of the peptides were produced by the Fmoc procedure in an automated bench top simultaneous multiple solid-phase peptide synthesizer (PSSM 8 system from Shimadzu, Tokyo, Japan). The final deprotected peptides were purified by HPLC (Shimadzu, Tokyo, Japan) in a semi-preparative column. The molecular mass and purity of the synthesized peptides were checked by Maldi-TOF and/or peptide sequencing with a PPSQ-23 protein sequencer (Shimadzu, Tokyo, Japan). The concentration of the Abz peptide solutions was determined by measuring the absorption of the 2,4-dinitrophenyl group at 365 nm (λ = 17,300 M^−1^ cm^−1^). The pERTKR-MCA (pE = pyroglutamic acid) was purchased from Peptanova (Sandhausen, Germany).

### Determination of hysteretic parameters

mPC1/3 activities were monitored spectrofluorometrically in a Hitachi F-2500 spectrofluorometer using the FRET peptides as substrates, with wavelengths of excitation at 320 nm and emission at 420 nm for Abz, and excitation at 380 and emission at 460 nm for MCA peptide. A standard cuvette (1 cm pathlength) containing 0.5 mL of substrate solution was placed in a thermostatically-controlled cell compartment for 5 min before the addition of enzyme. The kinetic parameters of peptide hydrolysis were determined in 20 mM BisTris pH 6.0 at 37°C, any other condition is indicated in the text. The pH values were adjusted at 25°C and checked before assays at 37°C using a model 710A Orion pH meter with an automatic temperature compensation (ATC) glass probe.

The reactions were monitored continuously based on the fluorescence of the released product. The time course of product generated by mPC1/3 cleavage was fitted to the exponential function Equation 1 using the software Grafit version 5.0 (Erithacus Software, Horley, Surrey, U.K.), and the hysteretic parameters (*k*, τ, v_ss_ and v_i_) were obtained.
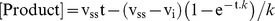
(1)where v_i_ is the initial velocity, v_ss_ is the steady-state velocity and *k* is the apparent rate constant for the transition between v_i_ and v_ss_. The secondary constant induction time *τ* (*k* ^−1^) can also be obtained. This parameter can be understood as the time required to reach the steady state. The rate of increase in fluorescence was converted into moles of substrate hydrolyzed per second based on the fluorescence curves of standard peptide solutions after total enzymatic hydrolysis. The model of hysteretic enzymes argued that the relationship of the steady-state velocity (v_ss_) *vs.* substrate may follow a Michaelian kinetics when appropriate [22]. The inner-filter effect was corrected using an empirical equation, and the kinetic parameters were calculated according to Wilkinson [23]. The enzyme concentration for initial rate determinations was chosen so that maximum 5% of the substrate was hydrolyzed.

Histatin-3 and Histatin-5 were synthesized by solid phase procedures as mentioned above with adding the fluorescence label Abz at N-terminus in order to improve the detection and quantification in HPLC-by fluorescence detector (LCMS-2010, Shimadzu, Tokyo, Japan).

The synthesized fluorescent labeled peptide Abz-DSHAKRHHGYKRKFHEKHHSHRGYR-NH_2_ (Abz-histatin-5) was used for calibration of HPLC-ESI-MS, since Histatin-5 is the product of Histatin-3 hydrolysis by PC1/3. Five different concentrations of Abz-histatin-5 ranging from 0.4 to 2.4 micromols were measured at Ex = 320 nm and Em = 420 nm. The linear fit of moles of Abz vs. Arbitrary Units of the Integrated Area (AUIA) gives a slope of 1.82×10^6^ AUIA/µmols of Abz-peptidyl, and this constant was used to quantify the Abz-Histatin-3 hydrolysis by mPC1/3. The kinetic analysis was performed using Abz-Histatin-3 concentration in the range of 1–20 µM. 20 nM of mPC1/3 were incubated with Abz-histatin-3 at each substrate concentration and aliquots were collected following the reaction time. The reactions were stopped with 5% TFA and 100 µl of each aliquot were analysed on HPLC-ESI-MS.

### Determination of peptide cleavage sites

The scissile bonds of hydrolyzed peptides were identified by the isolation of fragments using analytical HPLC followed by determination of their molecular mass by LCMS-2010 equipped with an ESI-probe (Shimadzu, Tokyo, Japan).

## Results

### Lag-phase analysis of mPC1/3 activity

We worked only with the purified homogeneous 66-kDa form in order to analyze mPC1/3 activity without further self-processing steps. The time course of hydrolysis with the distict lag-phase in the pre-steady-state (Figure 1) was also observed with the commercially available recombinant mPC1/3 66-kDa (R&D Systems, Minneapolis, MN, USA) and also with 87-kDa mPC1/3.

**Figure 3:**
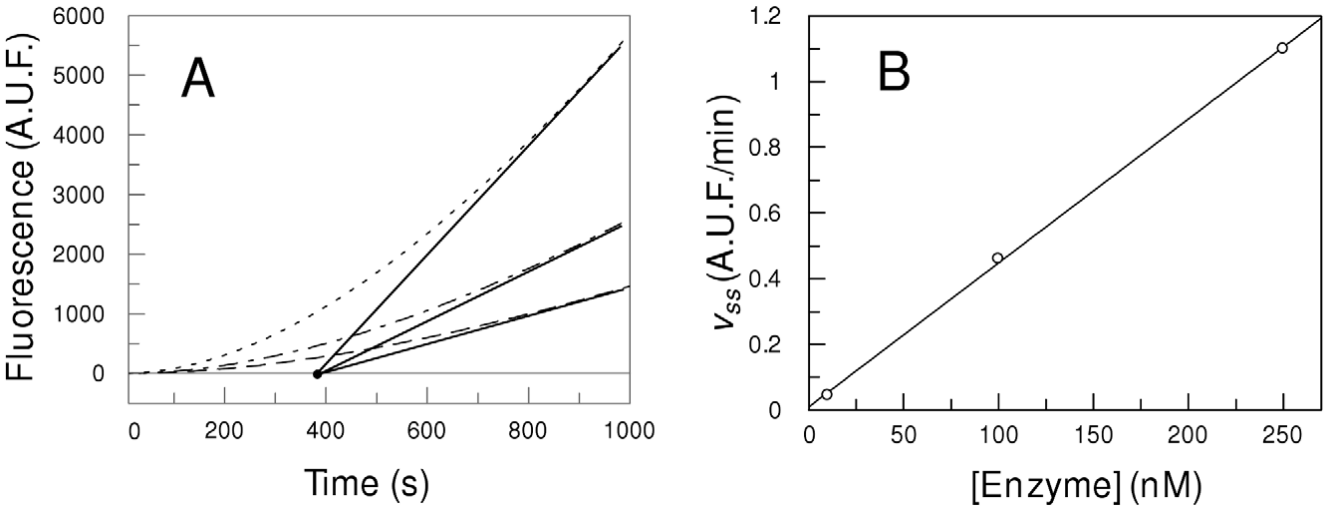
Effects of mPC1/3 concentration in time course of substrate hydrolysis. Panel A shows the progress curves of Abz-SKRSRRSVSV-Q-EDDnp (10 µM) hydrolysis by mPC1/3 at 10 nM (dashed line), 20 nM (dot-dashed line) and 30 nM (dotted line) nM. Solid lines represent the tangent at the steady-state velocity (v_ss_). The intercept at the *x*-axis is the induction periodwhich does not change with the enzyme concentration. Panel B shows the v_ss_ of Abz-SKRSRRSVSVQ-EDDnp (10 µM) hydrolysis by mPC1/3 at 10, 100 and 250 nM, indicating that the steady-state velocity is direct proportional to the enzyme concentration.

The transient lag phase of mPC1/3 activity was analyzed as an exponential increase of reaction velocity until a constant velocity was reached. The time course of the released product was fitted to an expression for a single exponential approach to steady-state (Equation 1). Figure 3-A shows the time course of Abz-SKRSRRSVSV-Q-EDDnp hydrolysis with three different enzyme concentrations, and that the obtained steady-state velocities are proportional to enzyme concentration. Abz-SKRSRRSVSV-Q-EDDnp was chosen to illustrate the results, but all the assayed substrates presented similar behavior. The range of enzyme concentrations shown in Figure 3-A demonstrate that the parameter τ (intercept of x axis) kept constant and the same results were achieved with a higher concentration of enzyme till 250 nM. These results exclude the possibility that the observed lag phase of mPC1/3 activity is due to a slow enzyme oligomerization and/or dissociation into monomers because the v_ss_ velocities are linearly proportional to enzyme concentration (Figure 3-B). It was recently published that mPC1/3 preparations present oligomers and, non-linear plots of activity vs. enzyme dilution were observed at higher enzyme concentrations [24], however, at the enzyme concentrations we used in the present work this phenomenon was not observed.

**Figure 4:**
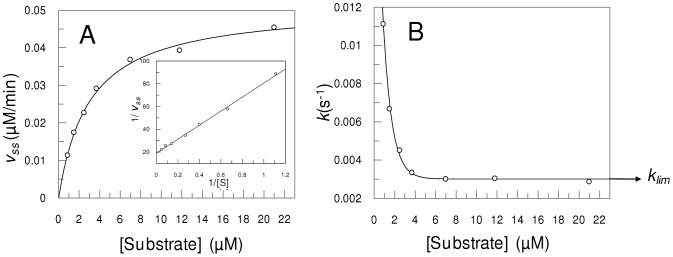
Substrate dependence of transient constants. Panel A indicates the v_ss_ vs. [S] plot and Lineweaver-Burk replot (inset). The substrate used was Abz-SKRSRRSVSVQ-EDDnp. Velocities were measured as product generated per minute (µM/min). Only steady-state velocities after the induction period were used. Panel B shows the *k*×[S] plot; the k constant decreases until a *k*_lim_ at higher substrate concentrations.

The time courses of FRET peptides and of pERTKR-MCA hydrolysis at various substrate concentrations were also obtained. The amounts of released products were fitted to Equation 1 and the parameters determined. The relationship of v_ss_ values with the concentration of Abz-SKRSRRSVSV-Q-EDDnp is shown in Figure 4-A, which demonstrates the hyperbolic behavior of the steady-state velocity obeying Michelis-Menten model and a linear Lineweaver-Burk plot (inset in Figure 4-A). The apparent rate constant (*k*) values that were determined at various concentrations of Abz-SKRSRRSVSV-Q-EDDnp are shown in Figure 4-B. It is noteworthy that as the substrate concentration rises, the *k* constants decrease from a high value until an asymptotic limit value (*k*_lim_) is reached, the reciprocal of which is the maximum induction period (τ_max_) at substrate saturation. Similar results were achieved with all other substrates assayed. Taken together, these results preclude any cooperativity effects on mPC1/3 hydrolytic activity because if cooperativity effects exist an increase in *k* with substrate concentration would be observed and also non linear Lineweaver-Burk plots would be observed.

Table 1 presents the kinetic constants obtained for all substrates assayed with mPC1/3. It is noteworthy that the magnitude of the apparent rate constants for the lag phase is on the order of minutes, whereas enzyme turnovers are on the order of seconds. This difference indicates that this transient approach to steady state cannot represent a step in the catalytic turnover. To support this conclusion we also performed the hydrolysis of FRET substrates by mPC1/3 in buffer with D_2_O (deuterium oxide) and H_2_O (water) as solvent. The induction time determined in D_2_O was equal to the obtained in H_2_O. However, the v_ss_ obtained in D_2_O was lower than the v_ss_ obtained in H_2_O (v_ss_^D2O^/v_ss_^H2O^ = 0.6). As we performed the assays with saturating substrate concentrations, the ratio v_ss_^D2O^/v_ss_^H2O^ represents the solvent isotope effect in the *k*_cat_ constant for the hydrolysis of the assayed substrate by mPC1/3. The observed value v_ss_^D2O^/v_ss_^H2O^ = 0.6 was approximately the same value reported for serine peptidases with deacylation as the rate-limiting step in the catalysis [25]. Therefore this solvent deuterium effect further supports the conclusion that the lag phase is not a *k*_cat_ step.

**Table 1:** Kinetic parameters for the hydrolysis of synthetic FRET peptides and pERTKR-MCA by 66-kDa mPC1/3.

Substrates	*k*_cat_ (s^−1^)	K_m_	*k*_lim_ (s^−1^)
Abz-SPREGKR↓SYSM-EDDnp	0.012±0.001	2.59±0.26	0.0026
Abz-YTPKSRR↓EVED-EDDnp	0.659±0.012	3.77±0.33	0.0029
Abz-SKRSRR↓SVSV-EDDnp	0.309±0.008	3.01±0.29	0.0029
Abz-DSHAKRHHGYKRKFHEKHHSHRGYR↓SNYLYDN-NH_2_ (Histatin 3)	1.253±0.094	2.32±0.34	0.0053
pERTKR↓MCA	0.030±0.002	17.82±2.34	0.0083

↓ indicates the cleavage site by 66-kDa mPC1/3 and ± standard errors are indicated.

In order to verify the effects of the reaction products on the lag phase, mPC1/3 was preincubated with the cleavage products Abz-SKRSRR and SVSVQ-EDDnp, before adding and recording the hydrolysis of the unbroken substrate Abz-SKRSRRSVSVQ-EDDnp. The results indicated no changes in the lag phase, excluding possible effects of the products of hydrolysis on the transient enzyme activation. We also analyzed the effects of lysine and arginine (100 mM) on the mPC1/3 time course, followed by other chemical agents (N-octyl glucosamine (0.1–1 mM), Brij (0.1–1%w/v), bovine serum albumin (0.1–1 mg/mL) and heparan sulfate (0.2 mM), and none of these additives affected the lag phase.

### Effect of Ca^2+^on lag-phase

**Figure 5:**
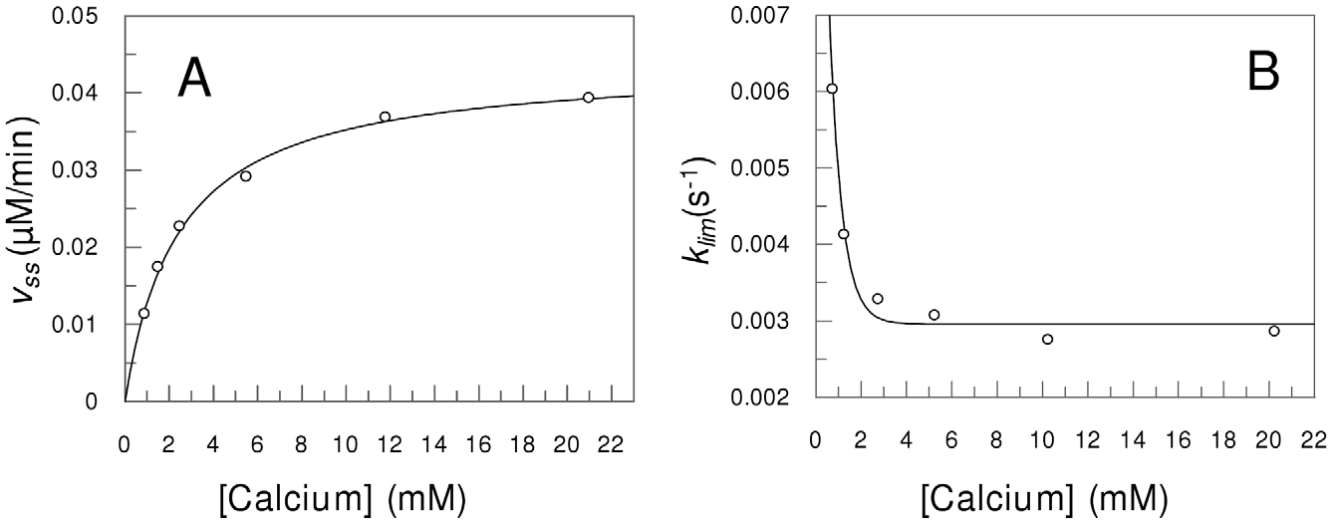
Effect of Ca^2+^ concentration on transient constants. Panel A shows the dependence of calcium on enzyme activity (v_max_). The substrate used was Abz-SKRSRRSVSVQ-EDDnp. Panel B shows the induction period as a function of the calcium concentration.

The effects of calcium on the parameters v_ss_, *k*, τ, K_m_, *k*_cat_ were also obtained in the calcium concentration range 1 mM to 20 mM. Figure 5-A shows that the v_ss_ vs. calcium concentration plot follows a saturating curve, and Figure 5-B shows that *k_lim_* values decrease with increasing calcium concentration, reaching an asymptotic limit value, whose reciprocal is the maximum induction period (τ_max_) at calcium saturation. The variation of the *k* parameter with calcium concentration indicates an allosteric effect of calcium on mPC1/3 and the structural details of this effect remain to be elucidated. The prototype enzymes Kex2 and furin that have crystallographic structures do not present this transient lag in the hydrolysis of their substrates [16], [26], [27].

### Effect of pH on lag-phase

**Figure 6:**
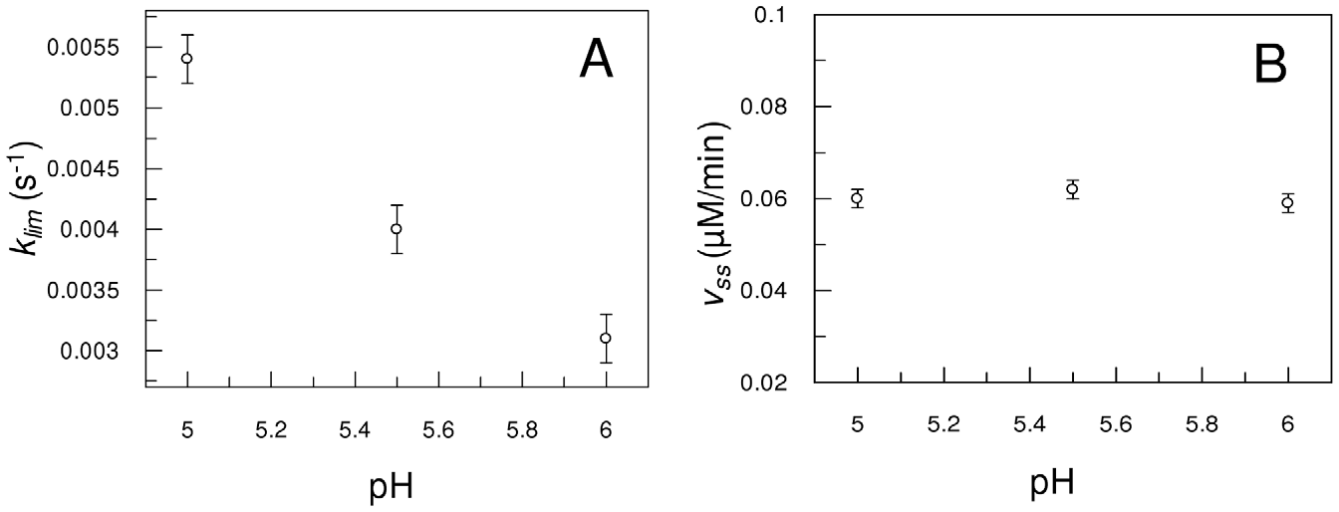
pH dependence of mPC1/3 steady-state velocity and *k*_lim_. Panel A shows the v_ss_ dependence with pH changes. Panel B shows the pH-dependence of the induction period of Abz-SKRSRRSVSVQ-EDDnp hydrolysis.

The dependency of lag phase with pH was examined with calcium and substrate under saturating concentrations, i.e., 20 mM calcium and 20 µM Abz-SKRSRRSVSVQ-EDDnp. This condition was used in order to avoid the interference of substrate or calcium ion concentration in the analysis of the pH-profile. The pH range was limited mainly due to the instability of the 66-kDa form at pH values outside the 5.0–6.0 range as earlier reported [15]. Despite the limited pH range, the effect of the pH on the lag phase was remarkable, as showed in Figure 6-A in contrast with the v_ss_ values that remained constant (Figure 6-B). The *k_lim_* is significantly higher in acidic pH showing that the induction period decreases (τ = *k* ^−1^) as the environment pH decreases from 7 to 5, consequently mPC1/3 reaches the v_ss_ faster at pH 5 than at pH 7.

### Hydrolysis of histatin-3 by PC1/3

This peptide with 32 amino acids was hydrolyzed exclusively at the R^25^-S bond (Abz-DSHAKRHHGYKRKFHEKHHSHRGYR^25^↓SNYLYDN). The quantification of the fluoresecent N-terminal labeled product of hydrolysis showed a clear a lag phase, confirming that also with a large substrate mPC1/3 presents a lag phase as observed for the short peptides. The parameters of the hydrolysis reaction are shown in Table 1. In contrast to the shorter substrates histatin-3 was hydrolyzed with significantly higher k_cat_ values, whereas the K_m_ values are of the same magnitude, except compared with pERTKR-MCA which was hydrolysed with K_m_ an order of magnitude higher.

## Discussion

The 66-kDa mPC1/3 exhibits substrate concentration dependent hysteresis that is found in enzymes involved in metabolic pathways, as earlier reviewed by Frieden [28]. This is an unprecedented observation in peptidases, but is frequent in regulatory enzymes with physiological relevance where this hysteretic behavior has been related to slow rate conformational changes in response to variations in the ligand concentration. In the regulatory enzymes the conformation changes represent the rate-limiting steps of their catalytic activities similar to that observed with mPC1/3, the lag phase of which is on the order of minutes in contrast to catalytic steps that are in seconds.

The lag phase parameter *k* varies between two limiting values with increasing substrate concentration (Figure 4-B), and this behavior indicates the existence of an equilibrium between active (*E′*) and inactive (*E*) states of the enzyme [22]. The slow transition of inactive to active mPC1/3 can be interpreted accordingly to the concept of hysteretic enzymes [29], [28], [22].

Considering the general model proposed by Frieden [28] for hysteretic enzymes we propose a mechamism that best describes mPC1/3 hydrolytic activity as showed in Scheme S1. The general model consists of two enzyme forms (*E* and *E′*) that bind to substrate (*E′S* and *ES′*), generating product by two ways. As the first-order *k* constant represents the slow transition between two different active forms of enzyme, *k* is a result of the constants between *E*↔*E′* (*k*_0_, *k*_−0_) and *ES*↔*E′S* (*k*_1_, *k*_−1_). Furthermore, *K_s_* is the affinity constant between *E*↔*ES* and *K′_s_* the affinity constant between *E′*↔*E′S*.

## Supporting Information

Scheme S1 In this model, the substrate bound rapidly to *E* and *E′*. As both *ES* and *E′S* were taken to be catalytically active, some degree of product formation was predicted immediately on mixing substrate with the enzyme. However, in our assays approximately zero product formation can be attributed to a selective binding of substrate to the active *E′* form, when most of the resting enzyme was in the *E* form [30]. Binding of substrate pulls progressively the enzyme population into the catalytically active form *E′*. Thus, most of the enzyme would have to undergo the slow hysteretic transition; when v_ss_ is achieved, equilibrium is reached between active and inactive enzyme. A simplified kinetic model derived from Scheme S1 can then be proposed (Scheme S2):
(TIF)

Scheme S2Therefore, Scheme S2 is simpler scheme that applies to the data of mPC1/3 activity on all assayed substrate, and the equation that represents this model is:
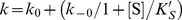
(2)Enzymes showing hysteretic behavior, particularly those with lag phase activation, exist mostly in the group of regulatory enzymes [31], [32], [30] that are involved in several physiological functions. PC1/3 is a pivotal protease in the processing of active peptides and hormones that are essential for normal mammalian development and survival [33]. The demonstration of hysteretic behavior for mPC1/3 indicating that this protease is highly regulated may be related to its important role in homeostatic and physiological processes. The dependence of the lag phase of activation of mPC1/3 on the concentrations of substrate and calcium (Figure 4 and 5), as well as on the pH (Figure 6), lends physiological relevance to this hysteretic behavior. As an example we can consider the available information on proinsulin processing to insulin [14], a process involving PC1/3. As proinsulin enters into secretory nascent granules, the pH decreases from 6.0 to 5.0 inside the granules, the calcium concentration rises from 0.1 mM to possible values >2 mM. The obtained results (Figures 5 and 6) showed that the pH and [Ca^2+^] variations can lead to large changes in mPC1/3 lag-activation. Then, PC1/3 hysteresis can be considered an additional control mechanism in the peptide hormone maturation processes as for instance in the transformation of proinsulin to insulin.(TIF)
