# Supplementary figures and images for: Hysteretic Behavior of Proprotein Convertase 1/3 (PC1/3)
Source: PLoS One. 2011 Sep 15;6(9):e24545. doi: 10.1371/journal.pone.0024545 (PMC3174183; doi:10.1371/journal.pone.0024545)

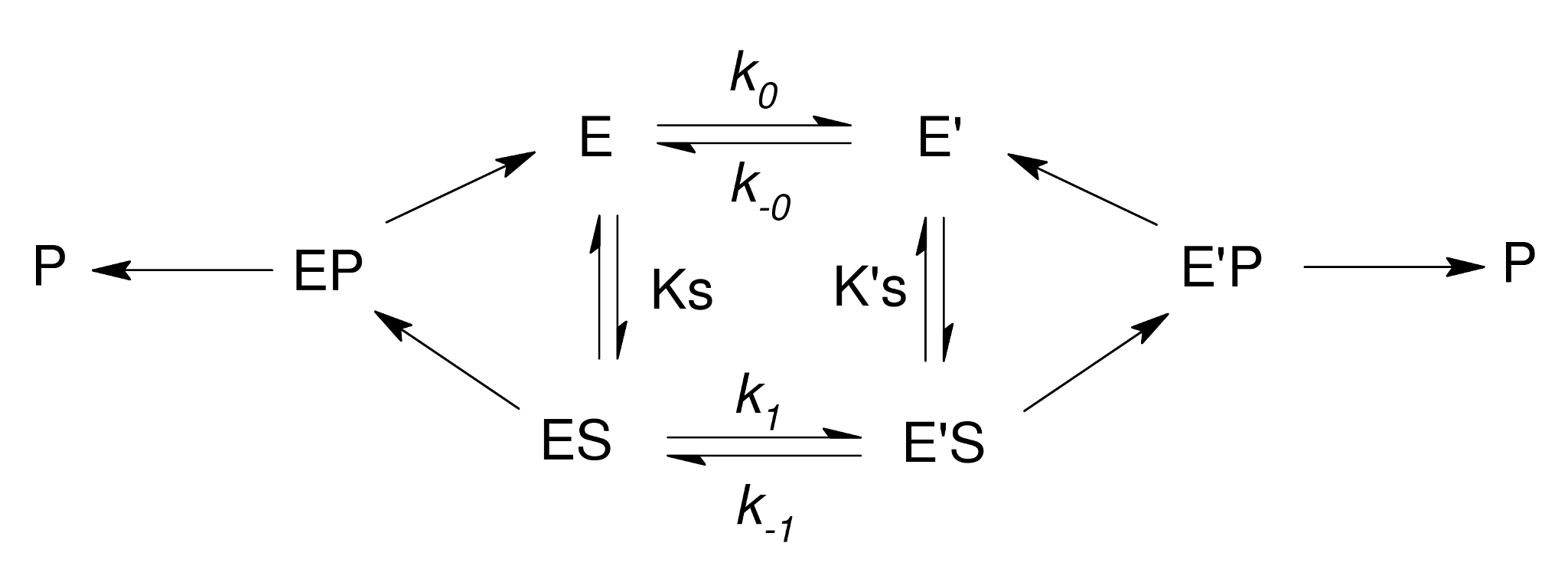

Supplement: Scheme S1 — In this model, the substrate bound rapidly to E and E′. As both ES and E′S were taken to be catalytically active, some degree of product formation was predicted immediately on mixing substrate with the enzyme. However, in our assays approximately zero product formation can be attributed to a selective binding of substrate to the active E′ form, when most of the resting enzyme was in the E form [30]. Binding of substrate pulls progressively the enzyme population into the catalytically active form E′. Thus, most of the enzyme would have to undergo the slow hysteretic transition; when vss is achieved, equilibrium is reached between active and inactive enzyme. A simplified kinetic model derived from Scheme S1 can then be proposed (Scheme S2): (TIF) [file pone.0024545.s001.tif]

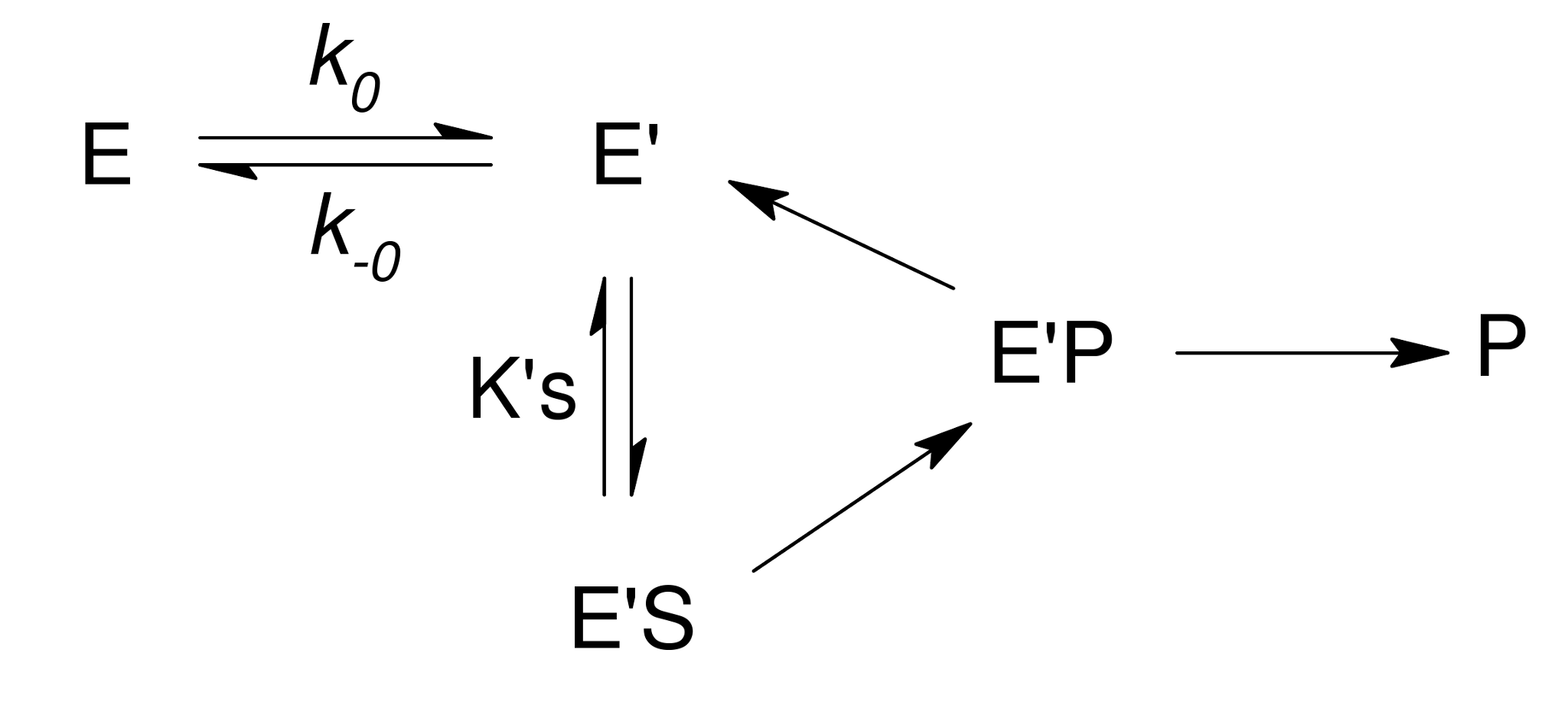

Supplement: Scheme S2 — Therefore, Scheme S2 is simpler scheme that applies to the data of mPC1/3 activity on all assayed substrate, and the equation that represents this model is:(2)Enzymes showing hysteretic behavior, particularly those with lag phase activation, exist mostly in the group of regulatory enzymes [31], [32], [30] that are involved in several physiological functions. PC1/3 is a pivotal protease in the processing of active peptides and hormones that are essential for normal mammalian development and survival [33]. The demonstration of hysteretic behavior for mPC1/3 indicating that this protease is highly regulated may be related to its important role in homeostatic and physiological processes. The dependence of the lag phase of activation of mPC1/3 on the concentrations of substrate and calcium (Figure 4 and 5), as well as on the pH (Figure 6), lends physiological relevance to this hysteretic behavior. As an example we can consider the available information on proinsulin processing to insulin [14], a process involving PC1/3. As proinsulin enters into secretory nascent granules, the pH decreases from 6.0 to 5.0 inside the granules, the calcium concentration rises from 0.1 mM to possible values >2 mM. The obtained results (Figures 5 and 6) showed that the pH and [Ca2+] variations can lead to large changes in mPC1/3 lag-activation. Then, PC1/3 hysteresis can be considered an additional control mechanism in the peptide hormone maturation processes as for instance in the transformation of proinsulin to insulin. (TIF) [file pone.0024545.s002.tif]
